# A novel Artificial Intelligence-based tool to assess anticholinergic burden: a survey

**DOI:** 10.1093/ageing/afac196

**Published:** 2022-08-27

**Authors:** Agostina Secchi, Hulkar Mamayusupova, Saber Sami, Ian Maidment, Simon Coulton, Phyo Kyaw Myint, Chris Fox

**Affiliations:** Kent and Medway NHS and Social Care Partnership Trust; AKFA University Medical School, Uzbekistan; University of Essex, CO4 3SQ, UK; University of East Anglia, Norwich, NR4 7TJ, UK; Aston University, Aston St, Birmingham B4 7ET, UK; University of Kent, Giles Ln, Canterbury CT2 7NZ, UK; Ageing Clinical & Experimental Research Team, Institute of Applied Health Sciences, University of Aberdeen, Aberdeen, Scotland, UK; University of Exeter, College of Medicine and Health

**Keywords:** anticholinergic, polypharmacy, adverse events, older people

## Abstract

**Background:**

many medications possess anticholinergic activity. Their use is associated with a number of serious adverse effects including cognitive effects. The cumulative anticholinergic effect of medications as assessed by tools such as the anticholinergic burden scale (AchB) can identify people particularly at risk of anticholinergic side-effects. Currently, >20 tools are available for clinicians to use, but there is no consensus on the most appropriate tool.

**Methods:**

a newly created online tool—International Anticholinergic Cognitive Burden Tool (IACT)—based on natural language processing and chemical structure analysis, was developed and made available for clinicians to test its functions. We carried out a survey (between 8th of February and 31st of March 2021) to assess the overall need for an assessment tool as well as the usability of the IACT.

**Results:**

a total of 110 responses were received from different countries and practitioners’ groups. The majority of the participants (86.11%) stated they would use a tool for AchB assessment if available and when they were asked to rate the IACT against other tools, amongst 34 responders, 20.59% rated it better and 8.82% rated it significantly better, 44.12% rated it neither better, nor worse, 14.71% rated it worse and 11.76% somewhat worse.

**Conclusion:**

there is a need for an anticholinergic burden calculator to assess the anticholinergicity of medications. Tools such as the IACT potentially could meet this demand due to its ability to assign scores to current and new medications appearing on the market based both on their chemical structure and reported adverse pharmacological effects.

## Key Points

- A novel Artificial Intelligence-based anticholinergic tool can be used to assess anticholinergic burden.
- The absolute necessity to evaluate anticholinergic burden when prescribing.
- The International Anticholinergic Cognitive Burden Tool can be easily deployed.

## Background and objectives

Global trends in use of medicines with anticholinergic activities are increasing [1–3]. In England, alone the use of anticholinergic medications or medications with anticholinergic activity has registered a significant increase between 1990 and 2001 (from 5.7 to 9.9%, respectively; [4]).

Anticholinergic activity is associated with a number of serious adverse events and it is often the result of prescribing multiple medications [5]. Reported adverse effects include dry mouth, nausea, constipation, blurred vision, urinary retention, cognitive impairment [6] and could increase risks of falls and may be associated with an increase in mortality [7–9]. Older people may be more susceptible to anticholinergic effects due to reduced renal and liver function, which affect the metabolism and elimination of the medications leading to increased exposure [10, 11]. There is also a linear relationship between anticholinergic burden and cardiovascular diseases or deaths [12].

Professionals should aim to reduce the overall anticholinergic burden (AchB) prescribed. Pharmacists might play an important role in deprescribing medications with anticholinergic activity [13]. To achieve this, an assessment of anticholinergic burden for individual medications is essential and it needs to be incorporated in routine clinical practice using a reliable scale.

Currently there are a number of scales available but National Institute for Health and Care [14] does not make recommendation of one over another and there is no gold standard scale [2]. Recent systematic reviews [15–17] could not recommend any particular tool. Lozano-Ortega *et al.* [18] identified 16 scales, 6 of which were suitable for quantification of anticholinergic exposure. However, the use of these scales and others currently in use [19] is limited, because they do not use an updating system, and there are differences in which medications are included and the impact of dose.

Against this background we developed a new method of measuring anticholinergic burden using machine learning technique—the International Anticholinergic Cognitive Burden (IACT) tool.

The novelty introduced with this tool is the use of a machine learning technique—a natural language processing—to develop an automated model available on a website portal. The anticholinergic burden is assessed by assigning a score based on reported adverse events and aligning closely with drug chemical structure, resulting in a more accurate and up-to-date scoring system.

The current report summarises the results of the survey we carried out with the view of testing the usability of this new calculator tool. The purpose was to better understand the benefits of usage as well as current limitations with the aim of future improved development.

## Methods

We developed a questionnaire using Qualtrics software (Qualtrics, Provo, UT, USA). The survey was first piloted among research team members with expertise in pharmacy, geriatric medicine, mental health and health service research who are involved in prescribing. After obtaining the ethical approval from University of East Anglia (reference: 2020/21-068) the survey was distributed via email and social media to various groups including NHS foundation trusts and pharmacies as well as internationally. Participants (doctors, non-medical prescribers, consultants, General Practitioners nurses and pharmacists) meeting the eligibility criteria, received the link to test the IACT and were invited to take part in the survey to evaluate the tool.

Participants were asked a mixture of closed and open-ended questions. Firstly, to gain more insight of their understanding of AChB calculation. Secondly, to ask an opinion on the usefulness of the IACT tool and possible suggestions for its improvement. The survey questions can be found in Appendix 2 (supplementary data are available in *Age and Ageing* online). More detailed explanation of the methods can be found in Appendix 4 (supplementary data are available in *Age and Ageing* online).

The feedback results were exported to Microsoft Excel and graphs plotted using Microsoft Excel (version 2020) and Origin (Pro) software (version 2021b, OriginLab Corporation, Northampton, MA, USA). The qualitative data extrapolated from the open-ended questions were analysed using a thematic analysis. This work was funded by EIRA (Enabling Innovation: Research to Application) at University of East Anglia and Research England and Eastern AHSN. Funders played no role in any parts of this work.

## Results

One hundred and ten professionals participated in this survey (Appendix 1, Panel A, Supplementary data are available in *Age and Ageing* online). In total, 73% were aware of national guidelines on AchB assessment and risk of cognitive impairment (Appendix 1, Panel B, Supplementary data are available in *Age and Ageing* online). Participants’ profession were 47.3% medical doctors (secondary and primary care), 38.2% pharmacists, 5.5% nurse prescribers and 9.1% other professions including physician associate, advance nurse practitioner and scientists (Appendix 1, Panel C, Supplementary data are available in *Age and Ageing* online).

When asked, the vast majority of 74.3% agreed that the prescriber should assess the AchB, whereas 20.2% responded as various professionals should be responsible and 5.5% were not sure (Appendix 1, Panel D, Supplementary data are available in *Age and Ageing* online).

**Figure 1:**
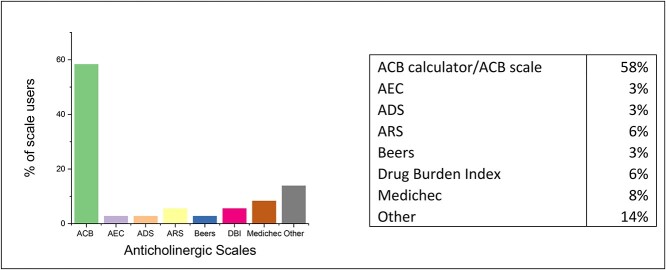
The usage of the different tools available to assess AchB. The total number of users *N* = 36. ACB, anticholinergic cognitive burden scale; AEC, anticholinergic effect on cognition scale; ADS, anticholinergic drug scale; ARS, anticholinergic risk scale; Beers, Beer’s scale; DBI, Drug Burden Index scale; Medichec, online tool.

The respondents were further asked whether they routinely assessed AchB and if yes which tools they frequently used. Around 54.13% answered affirmingly and the distribution of their tool usages is presented (Figure 1). Among those who used various tools, the majority (63.8%, total *N* = 36) used the ACB scale or ACB calculator. When asked to rate the usefulness of the tools (if used) in a Likert scale from 1 least helpful and 5 most helpful, among the 58 respondents, 42.86% of them scored 4, 31.43% scored 5 (mean = 4.027, 95% CI [3.75, 4.30]).

Participants were also asked to rate the new IACT tool against the tool they routinely used (Q10 of the survey) and of 34 responders, 20.59% rated it better and 8.82% a lot better, 44.12% rated it as neither better nor worse, whereas 14.71% rated it worse and 11.76% somewhat worse indicating the need for more education on the use of the tool.

Indeed, lack of knowledge was reported as a major barrier to the use of IACT (Q12).

Other barriers were time required and the need to include more medications with known and unknown AchB scores. According to participants, suggestions for alternatives, consideration of doses and co-morbidities would greatly enhance the usage of the tool. One of the main reasons, which prevented participants from assessing AchB scores were the lack of incorporated tools into the healthcare electronic record systems, or the impracticability of using the scoring tools when prescribing off-site (patients’ home).

## Discussion

The purpose of the survey was to obtain feedback on usage and benefits of newly created IACT tool when prescribing medications with anticholinergic activity.

In line with a previous studies [20], the results show an understanding of the importance of calculating AchB and interest in using a tool to calculate AchB in the routine clinical practice. However, the use of the tools was perceived as time consuming and more than one-third of the participants admitted not using them, indicating that more work should be done to simplify their use. The ACB scale seemed to be the most popular tool used to calculate the anticholinergic burden as easily accessible, although its limitations were acknowledged.

The participants were asked to rate the ACB and the other tools against our tool, the IACT. The new AChB calculation system introduced with the IACT, was perceived useful as based on characteristics such as chemical structure, medication side-effects and textual information allowing to score newly added medications in the market, hence differentiating from other tools [19]. Minor issues highlighted were taken in consideration for amendment. For example, we acknowledged the limitation of using the tool when working without internet access; we recognised that the use of the tool was time consuming for many doctors (Appendix 3, Supplementary data are available in *Age and Ageing* online). To overcome these limitations, we decided to make the IACT available in a web application (APP) accessible when internet access is limited. To facilitate the use of the tool we considered incorporating it within the prescribing web programme used in the GP surgeries such as SystmOne (TPP) so that medications with high levels of AChB are flagged up immediately when prescribing and prescribers do not need to enter the name of the medications in two different web programmes. Although the participants indicated that a tool to calculate AChB should be used by prescribers, the participants valued tools to calculate AChB and following future development our tool, the IACT, has the potential to fulfill this need [21].

Strengths. The survey was completed by professionals coming from a varied background, which helped with creating a greater validity of the data collected.

Limitations. Due to time constraints we could not recruit more international professionals who would have enriched the data and contributed to assess the usability of the IACT tool.

Implication for practice. The aim of this survey was to identify issues on the use of the IACT in clinical practice. The IACT, once refined, will help practitioners to standardise prescribing practice, it will help to improve medication monitoring and most importantly it will help to improve patients’ health by preventing anticholinergic side-effects [8, 22–24].

In summary, we conclude that machine learning based systems could be developed to quantify anticholinergic burden with the view of improving patient outcomes. IACT tool has the potential to help clinicians in their clinical decision around prescribing by providing an easy to access to up-to-date scoring system.

## Supplementary Material

aa-22-0172-File002_afac196
