# Supplementary material for: A novel Artificial Intelligence-based tool to assess anticholinergic burden: a survey
Source: Age Ageing. 2022 Aug 27;51(8):afac196. doi: 10.1093/ageing/afac196 (PMC9419503; doi:10.1093/ageing/afac196)
Supplement: aa-22-0172-File002_afac196 [file aa-22-0172-file002_afac196.docx]

**Manuscript Title**: A novel Artificial Intelligence (AI)-based tool to assess anticholinergic burden: a survey.

**Contents List**

Supplementary Figures:

Appendix 1: Survey results

Appendix 2: Survey Questions

Appendix 3: Results of Question 12 “What are the barriers to using the IACT tool?”

Appendix 4: Methods

**Supplementary Figures:**

**Appendix 1. Survey results**. Panel A: distribution of sample by world region. Panel B: awareness of guidelines on ACB assessment. Panel C: participants’ professional background. Panel D: perception of who is responsible for ACB assessment.

| 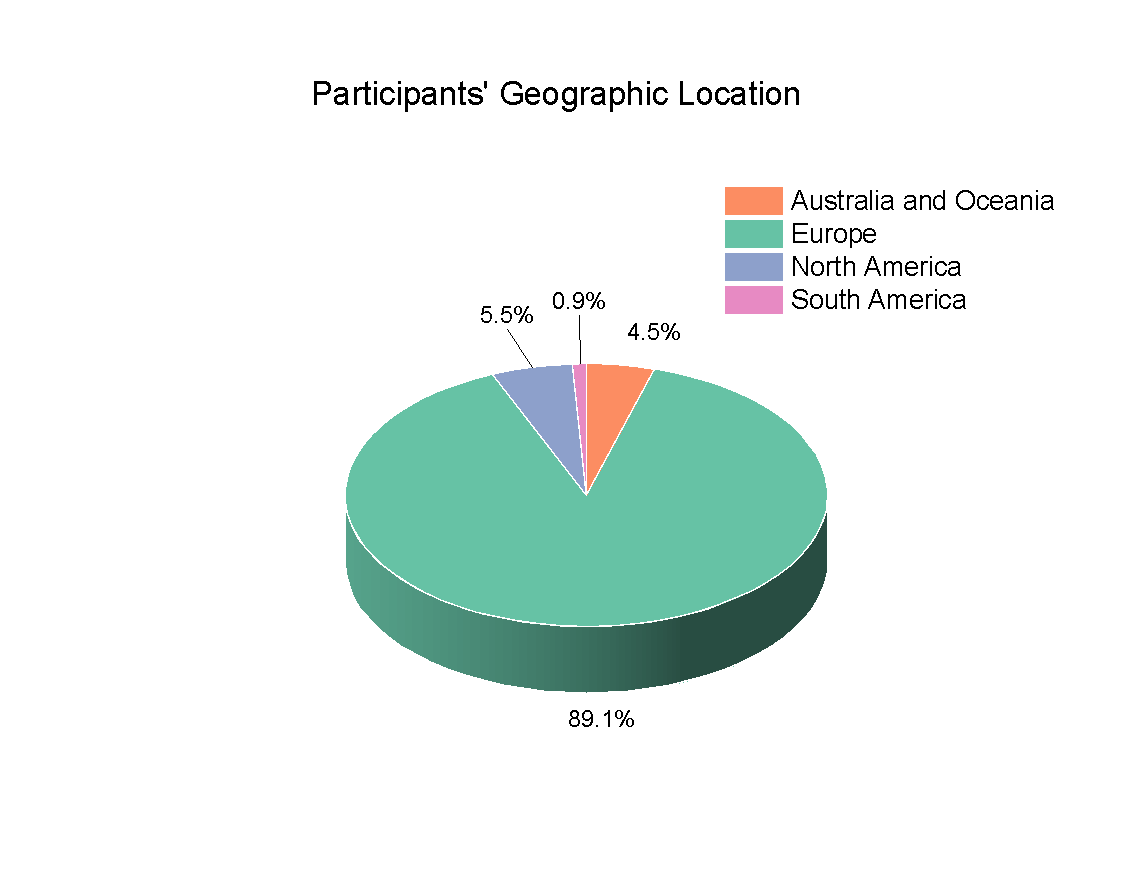  Panel A: total N=110 |   Panel B: total N=110 |
| --- | --- |
| 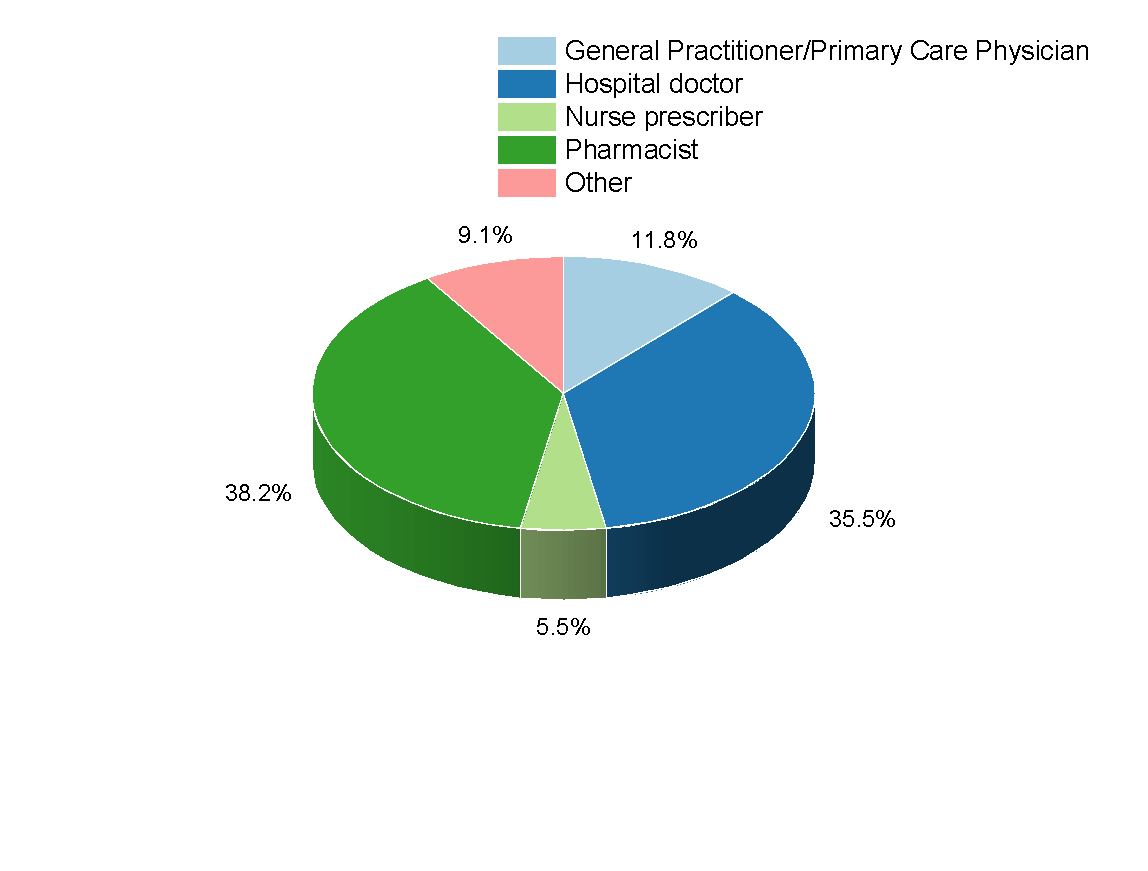  Panel C: total N=110 | 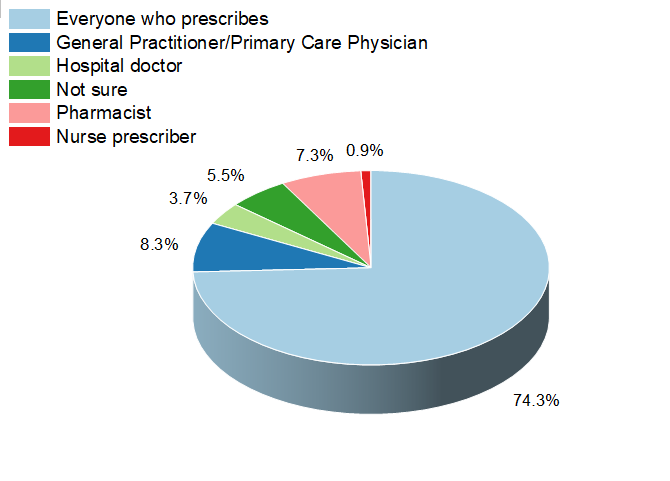  Panel D: total N=110 |

**Appendix 2. Survey Questions**

**Survey on usage of International Anticholinergic Burden (IACT) Web based tool**

Introduction Block:

Introduction

Older people are at risk of cognitive impairment due to the cumulative impact of medications with anticholinergic effect. In order to assist physicians, independent prescribers and pharmacists in identifying total anticholinergic burden, a pilot web-based “International Anticholinergic Burden” tool (IACT) was created. The purpose of this survey is to get feedback on usage and potential benefits of this newly created IACT tool when conducting medication assessments.

If you are interested in taking part, please click  [click here](https://essex.eu.qualtrics.com/CP/File.php?F=F_bwoYo2XgGR0wXhs) to read the Participant Information Sheet before clicking 'Next' to continue. Your responses to this survey are anonymous and will take around 10 minutes to complete.

1. If you agree with the following statements, please select "I agree" to each statement and press next to start the survey. (if not the survey ends)

- I understand my participation is voluntary and I can withdraw any time before submission
- I understand that to protect my anonymity any comments I submit should not include personal identifiable information
- I understand my survey data is anonymous and cannot be withdrawn after submission
- I agree to take part in this study

1. In which continent do you practice prescribing?

North America

South America

Europe

Asia

Australia and Oceania

Russia

Middle East

1. Are you aware of any National guidelines of Anticholinergic Burden (ACB) assessment to dementia and risk of dementia patients? For example, in the UK the Institute for Health and Care Excellence (NICE) has guidelines for Anticholinergic burden (ACB) assessment to dementia and risk of dementia patients.

Yes/No

1. What is your profession?

GP, Pharmacist, Nurse, Hospital Dr (specify your specialism), other (specify)

1. Whose role do you believe it is to conduct an ACB assessment? Please choose one

General Practitioner

Pharmacist

Nurse prescriber

Hospital specialist

Everyone who prescribes

I am not sure

1. Do you routinely assess anticholinergic burden?

Yes/No

if NO -go to “No block”

1. Do you currently use a tool to assess anticholinergic burden?

Yes/No

If NO, go to “No block”

1. Please state which tool you use

Free text entry

1. If you used a tool to assess ACB, please state how helpful you find the tool in clinical decision making, on a scale from 1 to 5. (5 = most helpful and 1= least helpful)

1 2 3 4 5

1. If you used the IACT tool, how would you rate it in terms of user interface compared to how you assess ACB at present. (5 = a lot better, 4=somewhat better, 3= neither better nor worse, 2= somewhat worse, 1= A lot worse)

1 2 3 4 5

==========Go to Q 13

“No” block (display only if No answered to Q5 or Q6)

1. Would you assess the Anticholinergic Burden if you had a tool?

Yes/No

1. What are the barriers to using the IACT tool?

Not a clinical priority

Prefer using another tool

Other (please specify) (free text entry) =========End the Survey

Final block (display directly after Q10)

1. What other information in the IACT tool would be useful to you?

Clinical significance of the ACB score

Suggestions for switching to alternative medicines

Other, please specify

=======End Survey

**Appendix 3. Results of Question 12 “What are the barriers to using the IACT tool?”**

**Appendix 4. Methods**

Eligibility: professionals from different background (doctors, pharmacists, nurses) involved in prescribing were invited to participate in a survey.

Design: a survey using Qualtrix software was developed after piloting with experts in the field. A mixed closed and open-ended questionnaire was developed. The open questions were used to elicit participants’ opinion on the IACT tool.

Recruitment/Distribution: the survey was sent to members of key professional groups including the British Geriatric Society, and the College of Mental Health Pharmacy. It was also circulated to staff in the NIHR Applied Collaboration East Midlands, the American Delirium Association, and Interdem. In addition, the survey was advertised by various social media platforms such as Twitter®.

Analysis: closed questions were analysed using Microsoft Excel (version 2020) and Origin (Pro) software. Answers to open-ended questions were analysed using thematic analysis.
